# An Exploratory Pilot Study of Coagulation- and Fibrinolysis-Related Proteins in Unexplained Infertility

**DOI:** 10.3390/ijms27135841

**Published:** 2026-06-28

**Authors:** Manjula Nandakumar, Abu Saleh Md Moin, Thozhukat Sathyapalan, Alexandra E. Butler, Stephen L. Atkin

**Affiliations:** 1 Research Department, Royal College of Surgeons in Ireland Bahrain, Adliya, Manama P.O. Box 15503, Bahrain; mnandakumar@rcsi.com (M.N.); amoin@rcsi.com (A.S.M.M.); satkin@rcsi.com (S.L.A.); 2 Academic Endocrinology, Diabetes and Metabolism, Hull York Medical School, Hull HU6 7RX, UK; thozhukat.sathyapalan@hyms.ac.uk

**Keywords:** unexplained infertility, male-factor infertility, plasma proteomics, coagulation, protein S, antithrombin-III, α2-antiplasmin, SOMAscan

## Abstract

Unexplained infertility (UI) accounts for an estimated 15–30% of infertility but its biological basis remains poorly defined. Plasma haemostatic abnormalities have been implicated in recurrent pregnancy loss; however, whether the circulating coagulation–fibrinolysis axis differs between women with UI and those with infertility attributable to other causes has not been characterised. The aim of this study was to compare 20 coagulation- and fibrinolysis-related proteins between women with UI and women with male-factor infertility (MFI) as control. In this exploratory case–control study, plasma from 11 women with UI and 14 women with MFI were analysed using SOMAscan proteomics. Group means were compared using Welch’s two-sample t-test, Cohen’s d with 95% confidence intervals, and a two-sided permutation test on the mean difference (20,000 random label permutations). False discovery rate (FDR) was used for multiple comparisons. Pairwise Pearson and Spearman correlations were performed. Three proteins differed between groups (*p* < 0.05), all lower in UI than in MFI: vitamin K-dependent protein S (Cohen’s d = −1.11, 95% CI −1.95 to −0.26; Welch *p* = 0.016; permutation *p* = 0.015), antithrombin-III (d = −1.00, 95% CI −1.84 to −0.16; *p* = 0.024; permutation *p* = 0.020) and α2-antiplasmin (d = −0.82, 95% CI −1.64 to 0.00; *p* = 0.051; permutation *p* = 0.054). No protein survived FDR correction. Within UI, the three proteins co-varied positively, with significant pairwise correlations between protein S and antithrombin-III (r = 0.67, *p* = 0.024) and between antithrombin-III and α2-antiplasmin (r = 0.71, *p* = 0.015). Within MFI, antithrombin-III and α2-antiplasmin remained tightly correlated (r = 0.70, *p* = 0.006) but protein S decoupled from both (r = 0.20 and 0.10, both non-significant). In this hypothesis-generating study, women with UI showed reduction in three plasma regulators of anticoagulant and antifibrinolytic capacity, protein S, antithrombin-III and α2-antiplasmin relative to women with MFI, raising the possibility that UI may be in part a subtle immunothrombotic/endothelial implantation disorder.

## 1. Introduction

Infertility is defined as the failure to achieve a clinical pregnancy after twelve months of regular, unprotected intercourse, which affects approximately one in six couples worldwide [1]. A diagnostic work-up identifies a discernible cause in the majority of these couples, including ovulatory disorders, tubal disease, endometriosis or male-factor abnormalities; in 15–30%, however, no abnormality is found and the diagnosis becomes one of exclusion, designated unexplained infertility (UI) [2,3]. UI is therefore a heterogeneous clinical category defined by the absence of a detectable defect on standard investigations rather than by a positive biological criterion, and the molecular basis for its persistence under assisted reproductive treatment remains poorly understood [3,4]. Male-factor infertility (MFI) affects about 7% of the male population and may be genetic, though other causes include endocrinopathies, cryptorchidism, exposure to gonadotoxins, obstruction and defective ejaculation [5]; female partners are clinically reproductively normal.

**Figure 1:**
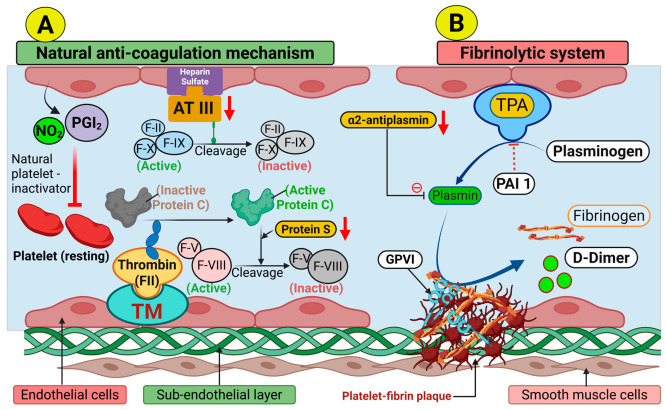
Endogenous anticoagulant and fibrinolytic mechanisms regulating platelet–fibrin clot formation and resolution. The schematic illustrates key endothelial and plasma pathways that maintain haemostatic balance by limiting platelet activation, suppressing coagulation factor activity, and promoting fibrin clot degradation. (**A**) Natural anticoagulation mechanisms: under physiological conditions, endothelial cells release nitric oxide (NO) and prostacyclin (PGI_2_), which inhibit platelet activation and help maintain platelets in a resting state. Antithrombin-III (ATIII), supported by endothelial heparan sulphate/heparin-like glycosaminoglycans, inhibits activated coagulation factors including thrombin/factor IIa, factor IXa, and factor Xa. In parallel, thrombin bound to endothelial thrombomodulin (TM) activates protein C. Activated protein C (APC), together with its cofactor protein S, proteolytically inactivates factors Va and VIIIa, thereby limiting further thrombin generation and clot propagation. (**B**) Fibrinolytic system: following platelet–fibrin plaque formation, endothelial tissue plasminogen activator (TPA) converts plasminogen into plasmin. Plasmin degrades the fibrin mesh within the clot, generating fibrin degradation products including D-dimer and contributing to clot resolution. This fibrinolytic pathway is negatively regulated by plasminogen activator inhibitor-1 (PAI-1), while α2-antiplasmin inhibits free plasmin activity and prevents excessive fibrinolysis. Downward red arrows indicate the decreased levels of the proteins. F, factor; GPVI, glycoprotein VI. Created with BioRender.com.

Successful implantation requires the highly coordinated invasion of trophoblast into the maternal decidua, the remodelling of the uterine spiral arteries and the establishment of a tightly regulated fibrin–fibrinolysis balance at the maternal–foetal interface [6]. The coagulation system contributes to each of these steps. Tissue factor and thrombin are expressed at the implantation site, where they participate not only in haemostasis but in trophoblast signalling and angiogenesis; regulated fibrinolysis is required to clear the transient fibrin scaffold that supports invasion [6,7]. From early gestation onward, maternal plasma acquires a measurably hypercoagulable phenotype—fibrinogen, factor VIII and von Willebrand factor rise while anticoagulants such as protein S decline—that is widely interpreted as an adaptation to the haemostatic challenge of placentation and delivery [8]; however, it is unclear if subtle disturbances of this maternal haemostatic balance can predispose to early reproductive failure. Inherited and acquired thrombophilias are associated with recurrent pregnancy loss, pre-eclampsia and foetal growth restriction linked to disorders of placenta dysfunction [9,10]. Potentially, at the earliest end of the same spectrum, defective implantation may manifest clinically not as recognised pregnancy loss but as failure to conceive [10]; it is reported that there is an excess of thrombophilic gene polymorphisms in women with UI [11]. The coagulation pathway is shown in Figure 1.

Coagulation plays a critical role throughout embryonic and foetal development and extends beyond its traditional function in haemostasis. Experimental studies have demonstrated that factor V contributes to embryonic vascular stability and thrombin-dependent angiogenesis, with deficiency resulting in developmental abnormalities and congenital bleeding phenotypes [12]. During foetal development, coagulation factor concentrations are generally lower than those observed in term neonates, although factor V levels are relatively preserved, suggesting a particular developmental importance [13]. Furthermore, while concentrations of factors VII and X increase with advancing gestation, factors II, V and VIII remain comparatively stable, with factors V and VIII maintained at higher levels than vitamin K-dependent coagulation factors [14]. This observation is notable given the direct relationship between factor V activity and thrombin generation, highlighting the importance of tightly regulated coagulation pathways in vascular development, placentation and successful pregnancy establishment [12,13,14,15].

We therefore conducted an exploratory case–control study to compare coagulation- and fibrinolysis-related proteins between women with UI and a control group of women with MFI, the latter clinically indicated as reproductively normal, with the null hypothesis that there would be no differences between these two groups.

## 2. Results

### 2.1. Patient Characteristics

There were no significant differences found between the MFI and UI groups in terms of age, body mass index (BMI), Homeostatic Model Assessment of Insulin Resistance (HOMA-IR) and serum lipids. C-reactive protein (CRP) and white blood cell (WBC) count, as indicators of inflammation and infection, were within a normal range and similar for both MFI and UI. There were no significant differences found between the MFI and UI groups with regard to levels of reproductive hormones, the number of positive pregnancy tests, eggs retrieved and embryos created, including the G3D3 which are the top-quality embryos at day 3 as per the Alpha consensus [16]. In addition, the fertility rates did not differ between the two groups (Table 1).

### 2.2. Cohort and Overall Plasma Proteomic Comparison

**Figure 2:**
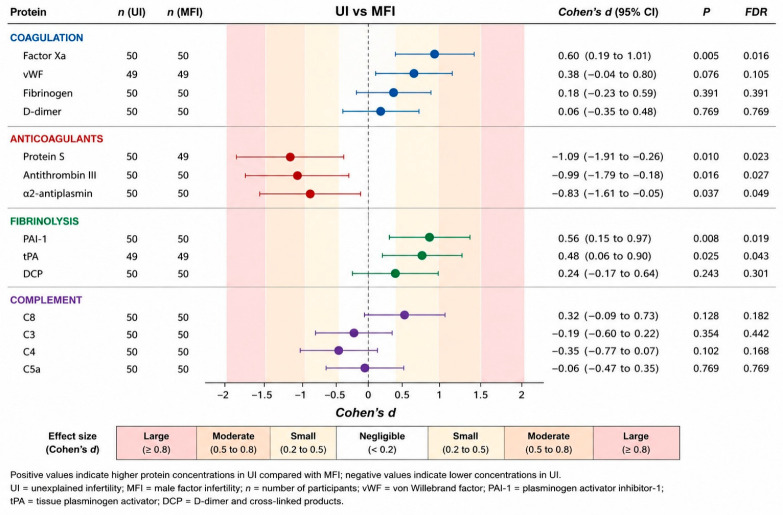
A forest plot of Cohen’s d as a diamond, the Hedges–Olkin 95% CI as a horizontal whisker bar, the proteins ordered by |d| descending so the largest effects sit at the top, a shaded |d| < 0.2 “trivial-effect” band, “← lower in UI/higher in UI →” direction labels under the axis, and a right-margin column printing d, the CI and the Welch *p*-value with significance stars. Red diamonds and bold y-labels mark the proteins reaching uncorrected Welch *p* < 0.05—protein S and antithrombin-III; α2-antiplasmin sits just on the edge with d = −0.82 [−1.64, 0.00] and is plotted in blue because its p shows a trend at 0.0509. Negative d means lower in UI (significance * *p* < 0.05). No protein survived FDR correction. PAI-1, plasminogen activator inhibitor-1.

Results across all 20 proteins, sorted by the magnitude of Cohen’s d, are reported in Table 2 and the forest plot shown in Figure 2. Three proteins differed between groups at the conventional uncorrected threshold (*p* < 0.05) on Welch’s test, and all three were lower in UI than in MFI: vitamin K-dependent protein S (UI 3590 ± 622 vs. MFI 4187 ± 466 RFU; Welch *p* = 0.016; Cohen’s d = −1.11, 95% CI −1.95 to −0.26; permutation *p* = 0.015), antithrombin-III (UI 113,349 ± 15,145 vs. MFI 127,511 ± 13,371 RFU; *p* = 0.024; d = −1.00, 95% CI −1.84 to −0.16; permutation *p* = 0.020), and α2-antiplasmin (UI 2044 ± 199 vs. MFI 2217 ± 219 RFU; *p* = 0.051; d = −0.82, 95% CI −1.64 to 0.00; permutation *p* = 0.054). Welch and permutation *p*-values agreed closely throughout the panel, indicating that normal-theory inference was not unduly affected by the small sample size. No protein survived FDR correction.

### 2.3. Correlations Amongst Protein S, Antithrombin-III and α2-Antiplasmin

**Figure 3:**
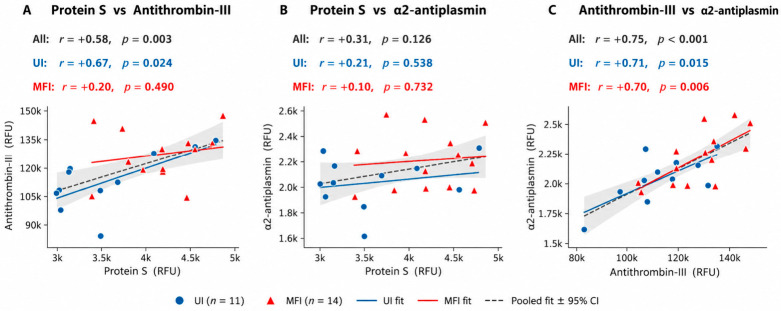
Pairwise Pearson correlations among three plasma anticoagulant proteins (protein S, antithrombin-III, α2-antiplasmin) in women with unexplained infertility (UI; blue circles, *n* = 11) and male-factor infertility (MFI; red triangles, *n* = 14). Solid coloured lines, within-group ordinary least-squares (OLS) fits; dashed grey line and shaded band, pooled OLS fit and its 95% confidence interval. Pearson r and two-sided *p*-values are reported above each panel for the pooled cohort (All). UI, unexplained infertility; MFI, male-factor infertility; RFU, relative fluorescent units.

Because these three proteins all reflect components of plasma anticoagulant control, we examined whether their levels co-varied within and between groups (Figure 3, Table 3). Pearson correlation coefficients with two-sided *p*-values were computed for each pair, both pooled across all 25 participants and separately within UI and MFI.

In the pooled cohort, antithrombin-III and α2-antiplasmin were strongly correlated (r = 0.75, 95% CI 0.51 to 0.88, *p* < 0.001), and protein S correlated moderately with antithrombin-III (r = 0.58, 95% CI 0.24 to 0.79, *p* = 0.003). The protein S–α2-antiplasmin association was weaker and did not reach significance (r = 0.31, 95% CI −0.09 to 0.63, *p* = 0.13). Spearman rank correlations gave concordant results (ρ = 0.67, 0.64 and 0.28 for the same three pairs).

### 2.4. Sensitivity Analysis

The leave-one-out maximum *p*-values across the 25 single-subject deletions were 0.035 for protein S and 0.048 for antithrombin-III, so significance was maintained in every leave-out (Supplementary Table S1).

## 3. Discussion

In this exploratory plasma proteomic comparison of women with UI and the MFI reference group, three of 20 coagulation-related proteins differed between groups at the conventional uncorrected threshold, and all three were lower in UI: vitamin K-dependent protein S, antithrombin-III, and α2-antiplasmin. The effect sizes were large (|d| = 0.82–1.11), the directionality was consistent, and within-group correlation analysis suggested that there may be coordinated regulation of these proteins in UI (all three proteins decreasing) with partial decoupling in MFI (only antithrombin-III decreasing), rejecting the null hypothesis. The sensitivity analysis supported the principal findings for protein S and antithrombin-III, qualifying the α2-antiplasmin result as suggestive rather than definitive. Together, these data suggest that there may be a reduction in plasma anticoagulant and antifibrinolytic capacity in UI [1], and indicate that the within-UI correlation pattern is a hypothesis-generating signal that requires confirmation in a larger cohort.

The three proteins identified here are not random members of the haemostatic panel; they represent three of the principal endogenous effectors within the plasma coagulation and fibrinolysis pathways as shown in Figure 1. Antithrombin is the dominant physiological inhibitor of thrombin and of the activated forms of factors IXa, Xa and Xia: constitutional or acquired antithrombin deficiency confers one of the strongest single-protein thrombotic risks [17]. Protein S, a vitamin K-dependent plasma cofactor, a non-enzymatic cofactor, accelerates the inactivation of factors Va and VIIIa by activated protein C and acts additionally as a cofactor for the tissue factor pathway inhibitor; protein S deficiency is similarly thrombophilic [18]. α2-antiplasmin is the principal physiological inhibitor of plasmin and the chief inhibitor of fibrinolysis; congenital α2-antiplasmin deficiency causes a severe bleeding phenotype rather than thrombosis, but the protein is otherwise a sensitive marker of the antifibrinolytic tone of plasma [19]. A potential coordinated reduction across all three may recalibrate the haemostatic balance toward a relatively pro-coagulant, fibrin-stabilising state, even in the absence of overt thrombophilia, with potentially relevant consequences for the fibrin dynamics of early implantation [6,9]. Historically, research on coagulation and reproduction has focused on inherited or acquired thrombophilia, particularly in the context of recurrent pregnancy loss and placental complications [20]. Anticoagulant proteins such as antithrombin-III, protein C and protein S not only limit thrombin generation but also modulate endothelial function and inflammatory signalling [21], processes that are increasingly recognised as integral to endometrial receptivity and early placentation [22] and consequently important in fertility and infertility. Thus, the direction of the observed changes provides insight into the functional consequences of this phenotype. Protein S and antithrombin-III are major endogenous anticoagulants that limit excessive thrombin generation, whereas α2-antiplasmin stabilises fibrin clots by inhibiting plasmin-mediated fibrinolysis. Collectively, the pattern observed in UI is compatible with a relative shift in haemostatic balance towards enhanced fibrin persistence and reduced fibrinolytic activity. Rather than indicating pathological thrombosis, this may reflect a subtle pro-coagulant and fibrin-stabilising environment at the maternal–foetal interface that could adversely affect implantation and early placental development. The present findings extend the literature in two ways: first, by showing that the potential imbalance may be detected at the plasma protein level rather than the germline genotype; and second, by implicating the antifibrinolytic regulator α2-antiplasmin in addition to the classical anticoagulant axis.

In UI, plasma protein S, antithrombin-III and α2-antiplasmin were positively correlated, and two of the three pairwise correlations reached nominal significance despite a sample size of eleven. In MFI, antithrombin-III and α2-antiplasmin remained tightly linked (r = 0.70), but protein S decoupled from both. Hepatocytes synthesise all three proteins, and several upstream pathways, including hepatic acute-phase signalling and oestrogen-responsive transcriptional programmes, are known to modulate plasma anticoagulants in concert [23]. This may mean that a coordinated drop, and tighter co-variation in UI, may be indicative of a shared upstream regulator for this group that would warrant further investigation.

Coagulation and fibrinolysis should not be considered simply as opposing independent processes, but rather as complementary mechanisms that together maintain haemostatic homeostasis, as is suggested in this study. Protein S and antithrombin-III regulate coagulation by limiting thrombin generation, whereas α2-antiplasmin regulates fibrinolysis through inhibition of plasmin-mediated fibrin degradation. Therefore, alterations in these proteins likely reflect changes in overall haemostatic balance. The observed profile in UI suggests a shift towards relative fibrin stabilisation rather than overt activation of coagulation alone, supporting the concept of a subtle implantation-associated haemostatic dysregulation.

The strength of this study is the use of the aptamer-based multiplexed proteomic platform that makes it possible to interrogate and undertake exploratory hypothesis-generating studies such as this one for dozens of plasma haemostatic proteins from a single sample, with reproducible sensitivity, in a way that would be difficult to assemble using traditional immunoassays. In addition, all subjects were in the same phase of their menstrual cycle. The study has several limitations including that the sample size was modest (UI *n* = 11, MFI *n* = 14), and although Welch and permutation *p*-values agreed and effect sizes were large, none of the marginal *p*-values survived FDR correction. This then would allow a robust study to be undertaken to measure both protein quantification and functionality at the same time. The cross-sectional design cannot establish causality; reduced anticoagulant levels might be a driver, a downstream consequence, or a marker of a shared third factor. The MFI reference group, while reproductively normal at the female level, cannot be considered a healthy general-population control and could itself differ from the unselected female norm. The SOMAscan platform quantifies protein abundance but does not assess functional activity, which is particularly relevant for enzyme-driven pathways such as coagulation; therefore, assays such as for functional antithrombin activity are needed. Additionally, the study population was ethnically homogeneous, and findings may not be generalisable to broader populations. Future studies should incorporate larger cohorts, functional assays of ATIII activity, and tissue-level analyses to validate and extend these findings. Finally, levels are reported in relative fluorescence units rather than international units; quantitative replication using standard immunoassays will be required in a larger robust study.

Whilst no inference for clinical practice can be given, it is intriguing that the three plasma proteins are already routinely measurable in clinical haematology laboratories, and a low-anticoagulant phenotype could in principle be detected with a panel that already exists for thrombophilia work-up. Further studies may be able to determine if such a phenotype identifies a subgroup of women with UI who might benefit from current interventions such as low-molecular-weight heparin, low-dose aspirin or combined regimens in the recurrent pregnancy loss literature [24].

In summary, in this exploratory comparison, women with UI showed coordinated reductions in three principal plasma regulators of coagulation and fibrinolysis (protein S, antithrombin-III and α2-antiplasmin) compared to an MFI reference group, raising the possibility that UI may be, in part, a subtle immunothrombotic and endothelial implantation disorder. The observed pattern of coagulation and fibrinolytic proteins suggests a haemostatic environment shifted toward relative fibrin stabilisation and reduced anticoagulant activity. The coordinated protein changes and inter-protein correlations further suggest regulation by common upstream pathways linking endothelial function, haemostasis and implantation biology.

## 4. Materials and Methods

### 4.1. Study Design and Participants

This exploratory cross-sectional case–control study was conducted at the in vitro fertilisation (IVF) unit, Hull, UK. Twenty-five women were recruited sequentially over a four-month period. Due to the exploratory nature of the study, no power calculation was carried out and a study population of 25 was determined based on participant availability and the inclusion/exclusion criteria applied. Ethical approval was granted by the Yorkshire and The Humber NRES Ethics Committee, UK (approval number 02/03/043) [25]. The study population was women diagnosed with UI, in accord with recognised guidelines [4] that included diagnostic laparoscopy. The control population was healthy women with MFI [4], where the men were diagnosed as infertile [26,27]. Exclusion criteria included: documented immunological or inflammatory disease, acute or chronic infection, hepatic or renal insufficiency, diabetes mellitus, body mass index (BMI) > 30 kg/m^2^, outside the 20–45-year range, and individuals not undergoing IVF treatment. Medical history review confirmed that none of the participants were taking prescription or over-the-counter medications, all were nonsmokers, and all had abstained from alcohol for more than six months. Women with UI underwent diagnostic laparoscopy as part of their evaluation, 8–12 weeks prior to the initiation of IVF treatment.

### 4.2. IVF Protocol

All participants commenced IVF treatment during the subsequent menstrual cycle using a short antagonist protocol seven days after the luteal blood test was taken. Recombinant follicle-stimulating hormone (rFSH) stimulation was initiated on day two of the cycle with either Merional (Pharmasure, Watford, UK) or Gonal-F (Merck Serono, Middlesex, UK). The dose was individualised according to Anti-Müllerian Hormone (AMH) levels, antral follicle count, age and ovarian response to prior treatment. From day six of stimulation, premature luteinizing hormone (LH) surge was prevented using Cetrotide (GnRH antagonist; Merck Serono, Middlesex, UK) at 0.25 mg/day. Final oocyte maturation was triggered when ≥2 leading follicles reached ≥18 mm, using either 0.5 mg Buserelin (Sanofi-Aventis, Frankfurt, Germany) or 5000–10,000 IU human chorionic gonadotrophin (hCG; Pregnyl, Merck Sharp & Dohme, Rahway, NJ, USA). Oocyte retrieval was performed transvaginally 36 h later. Luteal phase support was initiated on the day of oocyte retrieval with vaginal progesterone (Uterogestan, Besins Iscovesco Laboratories, Paris, France; 600 mg nightly). Embryo transfer was carried out on day three or preferably day five (blastocyst stage) to maximise implantation potential. Embryos were graded at both cleavage and blastocyst stages using standardised morphological criteria [16].

### 4.3. Sample Collection

At the time of mock embryo transfer, ovulation was detected using transvaginal ultrasonography which confirmed the presence of the corpus luteum and at day 21 of the menstrual cycle, before initiation of IVF treatment and before administration of any hormonal therapy that was started at the next menstrual cycle. Venesection was undertaken with a 21-gauge needle and placed into tubes with Ethylenediaminetetraacetic acid (EDTA) anticoagulant. Plasma fasting blood samples were collected, centrifuged (3500× *g* for 15 min at 4 °C), and stored at −80 °C until analysis.

### 4.4. Biochemical and Hormonal Assays

#### 4.4.1. Metabolic Measures

Fasting blood glucose (FBG) was measured using a Synchron LX20 analyser (Beckman Coulter, High Wycombe, UK). Total cholesterol, triglycerides, and high-density lipoprotein cholesterol (HDL-c) were measured enzymatically using a Synchron LX20 analyser (Beckman Coulter). Low-density lipoprotein cholesterol (LDL-c) was calculated using the Friedewald equation [28]. Serum insulin concentrations were determined by competitive chemiluminescent immunoassay (Immulite 2000, Euro/DPC, Llanberis, UK). Insulin resistance was assessed using the Homeostatic Model Assessment of Insulin Resistance (HOMA-IR), calculated as (insulin × glucose)/22.5 [29]. Glycated haemoglobin (HbA1c) was determined by ion-exchange chromatography (Abbot Diagnostics, Maidenhead, UK). C-reactive protein (CRP) was evaluated using enzymatic assays on the Synchron LX20 analyser (Beckman Coulter). White blood cell count (WBC) was measured with a Beckman Coulter counter (Beckman Coulter).

#### 4.4.2. Reproductive Hormones

AMH levels were determined with an immunoenzymatic assay (Beckman Coulter). Circulating androgens were quantified by liquid chromatography–tandem mass spectrometry (LC/MS/MS; Acquity UPLC-Quattro Premier XE-MS, Waters, Manchester, UK). Sex hormone-binding globulin (SHBG) was assessed using an immunometric fluorescence assay (Immulite 2000 analyser; upper limit 2.0 nmol/L). The free androgen index (FAI) was calculated according to the formula (testosterone/SHBG) × 100. Thyroid hormone levels, thyroid stimulating hormone (TSH), free triiodothyronine (Free-T3), and free thyroxine (Free-T4), were determined using an immunoassay on the Abbott Architect i4000 platform (Abbott Diagnostics, Ciudad de México, Mexico).

### 4.5. Coagulation Protein Quantification

Coagulation plasma proteins were quantified using the Slow Off-rate Modified Aptamer (SOMAscan) platform, version 3.1 (SomaLogic, Boulder, CO, USA), as previously described [30,31]. In brief, synthetic SOMAmers with fluorescent labelling were first bound to analyte/primer beads, and the resulting complexes were immobilised on a streptavidin matrix. Ultraviolet (UV) light was applied to cleave the photocleavable linker, releasing the analyte–SOMAmer complexes into solution. These complexes were subsequently re-immobilised on a streptavidin matrix through analyte-mediated biotinylation, after which the SOMAmers were eluted and used as surrogates for protein quantification. Quantification was achieved through hybridization with complementary oligonucleotides, enabling accurate signal detection. Calibration standards were included, and normalisation procedures—comprising hybridization control, median signal scaling and calibration signal correction—were applied in accordance with established protocols for fibrinogen, antithrombin-III, D-dimer, coagulation factor Xa, coagulation factor X1, von Willebrand factor, alpha-2-antiplasmin, thrombin, protein C and S, plasminogen, tissue plasminogen activator and plasminogen activator inhibitor-1 (PAI-1).

### 4.6. Statistical Analysis

As this was an exploratory pilot, no formal power calculation was performed, but *n* = 25 was selected to estimate effect sizes for future studies: analyses were framed around effect-size estimation rather than power-driven hypothesis testing. Continuous variables are summarised as mean ± standard deviation. Between-group differences for each of the 20 plasma proteins were assessed with Welch’s two-sample t-test, allowing unequal variances. Effect sizes were quantified by Cohen’s d with two-sided 95% confidence intervals (CIs). To reduce reliance on the normality assumption at small sample size, every contrast was triangulated with a two-sided permutation test on the observed mean difference (20,000 random label permutations). Correction for multiple testing across the 20 proteins was performed using the Benjamini–Hochberg false discovery procedure (FDR). Pearson and Spearman pairwise correlations were computed pooled across the cohort (*n* = 25) and within each group (UI, *n* = 11; MFI, *n* = 14). Sensitivity analysis was performed to assess the robustness of the findings against small-sample artefacts: leave-one-out (LOO) diagnostics were applied to the three differentially altered proteins by removing each of the 25 subjects in turn and recomputing the mean, Welch’s t-statistic, two-sided *p*-value, Cohen’s d and its 95% confidence intervals. All analyses were performed in SPSS version 31.0.1.0 (IBM SPSS, Armonk, NY, USA) and R software (2023.06.2-561).

## Figures and Tables

**Table 1:** Demographic, biochemical data and in vitro fertilisation parameter data compared between male-factor infertility (*n* = 14) and unexplained infertility (*n* = 11). The data is presented as mean (SD). The *p* values were calculated using Welch’s t-test to determine differences between the groups.

	MFI	UI	*p* Value
Age (years)	32.6 ± 4.0	33.8 ± 5.3	0.51
BMI (kg/m^2^)	25.7 ± 2.6	25.3 ± 4.9	0.84
HOMA-IR	1.5 ± 0.7	1.9 ± 1.4	0.45
Cholesterol (mmol/L)	4.8 ± 0.8	4.6 ± 0.7	0.44
Triglycerides (mmol/L)	0.9 ± 0.5	1.0 ± 0.4	0.67
HDL-c (mmol/L)	1.6 ± 0.4	1.6 ± 0.2	0.97
LDL-c (mmol/L)	2.8 ± 0.7	2.4 ± 0.4	0.24
CRP (mg/L)	1.9 ± 1.3	2.4 ± 2.0	0.51
WBC × 10^9^/L	5.9 ± 1.7	7.2 ± 2.2	0.12
AMH (ng/mL)	22.4 ± 15.3	24.5 ± 12.5	0.72
FAI	1.4 ± 0.7	0.8 ± 0.9	0.11
TSH (mU/L)	2.3 ± 1.2	2.0 ± 0.8	0.53
Free-T3 (pmol/L)	4.7 ± 0.7)	4.8 ± 0.6	0.66
Free-T4 (pmol/L)	11.2 ± 1.6	11.4 ± 0.9	0.66
Positive pregnancy test	0.3 ± 0.5	0.3 ± 0.5	0.95
Number of eggs retrieved	9.0 ± 7.5	8.4 ± 3.2	0.78
Number of embryos created	3.7 ± 3.0	5.2 ± 2.4	0.18
G3D3	3.4 ± 2.2	2.7 ± 2.6	0.49
Fertility rate	0.6 ± 0.2	0.6 ± 0.4	0.89
Top-quality embryo ± proportion)	0.3 ± 0.2	0.4 ± 0.4	0.36

MFI, male-factor infertility; UI, unexplained infertility; BMI, body mass index; HOMA-IR, Homeostatic Model Assessment of Insulin Resistance; HDL-c, high-density lipoprotein cholesterol; LDL-c, low-density lipoprotein cholesterol; CRP, C-reactive protein; WBC, white blood cell count; AMH, Anti-Müllerian Hormone; FAI, free androgen index; TSH, thyroid stimulating hormone; Free-T3, free-triiodothyronine; Free-T4, free-thyroxine; G3D3, top-quality day 3 embryos.

**Table 2:** Group comparison of 20 plasma coagulation-related proteins between women with unexplained infertility (UI, *n* = 11) and male-factor infertility (MFI, *n* = 14). Mean ± SD are shown for each group. Welch’s two-sample t-statistic, Cohen’s d (pooled SD) with Hedges–Olkin 95% confidence interval, and two-sided permutation *p*-value (20,000 permutations) are reported. Rows are ordered by descending |Cohen’s d|.

Protein	UI Mean ± SD	MFI Mean ± SD	*p* (Welch)	Cohen’s d (95% CI)	*p* (Perm.)
Protein S	3590 ± 622	4187 ± 466	0.016	−1.11 (−1.95, −0.26)	**0.015**
Antithrombin-III	113,349 ± 15,145	127,511 ± 13,371	0.024	−1.00 (−1.84, −0.16)	**0.020**
α2-antiplasmin	2044 ± 199	2217 ± 219	0.051	−0.82 (−1.64, +0.00)	0.054
Thrombin	620 ± 251	749 ± 142	0.150	−0.65 (−1.46, +0.16)	0.116
Coagulation factor Xa	5347 ± 821	4913 ± 624	0.163	+0.61 (−0.20, +1.41)	0.145
PAI-1	334 ± 227	225 ± 160	0.193	+0.57 (−0.24, +1.37)	0.172
P-selectin	16,620 ± 11,235	13,288 ± 3141	0.360	+0.43 (−0.37, +1.23)	0.360
von Willebrand factor	9222 ± 4813	7008 ± 6708	0.347	+0.37 (−0.42, +1.17)	0.381
Fibronectin fragment 3	3119 ± 2145	2528 ± 2540	0.535	+0.25 (−0.54, +1.04)	0.523
Heparin cofactor II	3988 ± 953	3807 ± 592	0.588	+0.24 (−0.56, +1.03)	0.563
Angiostatin	28,076 ± 7664	26,366 ± 7859	0.589	+0.22 (−0.57, +1.01)	0.595
Fibronectin	17,240 ± 14,789	14,135 ± 15,163	0.612	+0.21 (−0.58, +1.00)	0.523
Fibrinogen	179,174 ± 27,352	174,279 ± 25,553	0.652	+0.19 (−0.61, +0.98)	0.652
Fibrinogen γ chain	179,174 ± 27,352	174,279 ± 25,553	0.652	+0.19 (−0.61, +0.98)	0.650
Fibronectin fragment 4	64,569 ± 32,765	59,279 ± 33,450	0.695	+0.16 (−0.63, +0.95)	0.647
Tissue factor	1008 ± 597	940 ± 266	0.732	+0.15 (−0.64, +0.94)	0.725
Prothrombin	149,456 ± 16,182	151,512 ± 17,269	0.762	−0.12 (−0.91, +0.67)	0.766
D-dimer	13,789 ± 2971	13,615 ± 2828	0.883	+0.06 (−0.73, +0.85)	0.882
Coagulation factor XI	1677 ± 301	1687 ± 272	0.934	−0.03 (−0.82, +0.76)	0.932
Plasma kallikrein	21,377 ± 3201	21,328 ± 4408	0.974	+0.01 (−0.78, +0.80)	0.974

Values are mean ± SD in relative fluorescence units (RFUs). Welch t = two-sample t-statistic allowing unequal variances p (Welch) is two-sided. Cohen’s d uses the pooled standard deviation; 95% CI by the Hedges–Olkin large-sample formula. *p* (perm.) is the two-sided permutation *p*-value on the mean difference with 20,000 random label permutations (seed = 20,260,502). Bold rows: *p* (Welch) < 0.05. Negative d indicates lower abundance in UI. After FDR correction across 20 tests, no protein survived at q = 0.05 (smallest adjusted q ≈ 0.24). PAI-1, plasminogen activator inhibitor-1; UI, unexplained infertility; MFI, male-factor infertility.

**Table 3:** Pairwise Pearson and Spearman correlations among protein S, antithrombin-III and α2-antiplasmin, computed across the pooled cohort (All, *n* = 25) and within each group (UI, *n* = 11; MFI, *n* = 14). For each pair, the correlation coefficient is reported with its Fisher z-transformed 95% confidence interval and two-sided *p*-value (df = n − 2).

Group	n	Variable Pair	Pearson r (95% CI)	*p*	Spearman ρ (95% CI)	*p*
All	25	Protein S vs. Antithrombin-III	+0.58 (+0.23, +0.79)	0.003	+0.64 (+0.32, +0.82)	**<0.001**
All	25	Protein S vs. α2-antiplasmin	+0.31 (−0.09, +0.63)	0.126	+0.28 (−0.13, +0.61)	0.181
All	25	Antithrombin-III vs. α2-antiplasmin	+0.75 (+0.50, +0.88)	<0.001	+0.67 (+0.38, +0.84)	**<0.001**
UI	11	Protein S vs. Antithrombin-III	+0.67 (+0.12, +0.91)	0.024	+0.65 (+0.09, +0.90)	**0.029**
UI	11	Protein S vs. α2-antiplasmin	+0.21 (−0.45, +0.72)	0.538	+0.14 (−0.50, +0.68)	0.689
UI	11	Antithrombin-III vs. α2-antiplasmin	+0.71 (+0.19, +0.92)	0.015	+0.66 (+0.11, +0.90)	**0.026**
MFI	14	Protein S vs. Antithrombin-III	+0.20 (−0.37, +0.66)	0.490	+0.30 (−0.28, +0.71)	0.303
MFI	14	Protein S vs. α2-antiplasmin	+0.10 (−0.45, +0.60)	0.732	+0.03 (−0.51, +0.55)	0.911
MFI	14	Antithrombin-III vs. α2-antiplasmin	+0.70 (+0.26, +0.90)	0.006	+0.58 (+0.07, +0.85)	**0.030**

Pearson r and Spearman ρ are reported with their Fisher z-transformed 95% confidence intervals; two-sided *p*-values are based on the t-statistic r√[(n − 2)/(1 − r^2^)] with df = n − 2. Bold rows: Pearson *p* < 0.05. UI, unexplained infertility; MFI, male-factor infertility.

## Data Availability

The data that support the findings of this study are available from the corresponding author, A.E.B., upon reasonable request.

## Supplementary Materials

The following supporting information can be downloaded at: https://www.mdpi.com/article/10.3390/ijms27135841/s1.
