# Supplementary material for: An Exploratory Pilot Study of Coagulation- and Fibrinolysis-Related Proteins in Unexplained Infertility
Source: Int J Mol Sci. 2026 Jun 28;27(13):5841. doi: 10.3390/ijms27135841 (PMC13360802; doi:10.3390/ijms27135841)
Supplement: Supplementary file 1 [file ijms-27-05841-s001.zip › ijms-4385182-supplementary.pdf]

Supplementary Table S1. Leave-one-out (LOO) sensitivity for the three Welch-significant proteins. Each of the 25 subjects was removed in turn and Welch's t-test was repeated. Reported are the observed (base) statistics and the minimum / maximum across all 25 LOO replicates.

Protein	Statistic	Base	LOO min	LOO max	Always $p < .05$
**Protein S**	Cohen's d	-1.11	-1.47	-1.00	—
	Welch p	.016	.002	.035	**Yes**
	Welch t	-2.65			
**Antithrombin-III**	Cohen's d	-1.00	-1.19	-0.87	—
	Welch p	.024	.010	.048	**Yes**
	Welch t	-2.44			
**α2-antiplasmin**	Cohen's d	-0.82	-0.97	-0.67	—
	Welch p	.051	.026	.096	No
	Welch t	-2.06			

Reported are the observed (Base) statistic and the minimum and maximum value across the 25 single-subject leave-outs. "Always p < .05" indicates whether the Welch two-sided p-value remained below 0.05 in every leave-out. Cohen's d is on the pooled-SD scale with the convention d = mean(UI) − mean(MFI).
